# Inhibition of fibroblast secreted QSOX1 perturbs extracellular matrix in the tumor microenvironment and decreases tumor growth and metastasis in murine cancer models

**DOI:** 10.18632/oncotarget.27438

**Published:** 2020-01-28

**Authors:** Tal Feldman, Iris Grossman-Haham, Yoav Elkis, Patrick Vilela, Neta Moskovits, Iris Barshack, Tomer M. Salame, Deborah Fass, Tal Ilani

**Affiliations:** ^1^Department of Structural Biology, Weizmann Institute of Science, Rehovot 7610001, Israel; ^2^Almog Diagnostic, Shoham 6081513, Israel; ^3^Felsenstein Medical Research Center, Sackler Faculty of Medicine, Tel Aviv University, Tel Aviv 6997801, Israel; ^4^Institute of Pathology, Sheba Medical Center Tel Hashomer, Sackler Faculty of Medicine, Tel Aviv University, Tel Aviv 6997801, Israel; ^5^Life Sciences Core Facilities, Weizmann Institute of Science, Rehovot 7610001, Israel

**Keywords:** monoclonal antibody inhibitor, tumor microenvironment, fibroblasts, extracellular matrix remodeling, disulfide bonds

## Abstract

Extracellular matrix (ECM) plays an important role in tumor development and dissemination, but few points of therapeutic intervention targeting ECM of the tumor microenvironment have been exploited to date. Recent observations suggest that the enzymatic introduction of disulfide bond cross-links into the ECM may be modulated to affect cancer progression. Specifically, the disulfide bond-forming activity of the enzyme Quiescin sulfhydryl oxidase 1 (QSOX1) is required by fibroblasts to assemble ECM components for adhesion and migration of cancer cells. Based on this finding and the increased QSOX1 expression in the stroma of aggressive breast carcinomas, we developed monoclonal antibody inhibitors with the aim of preventing QSOX1 from participating in pro-metastatic ECM remodeling. Here we show that QSOX1 inhibitory antibodies decreased tumor growth and metastasis in murine cancer models and had added benefits when provided together with chemotherapy. Mechanistically, the inhibitors dampened stromal participation in tumor development, as the tumors of treated animals showed fewer myofibroblasts and poorer ECM organization. Thus, our findings demonstrate that specifically targeting excess stromal QSOX1 secreted in response to tumor-cell signaling provides a means to modulate the tumor microenvironment and may complement other therapeutic approaches in cancer.

## INTRODUCTION

Tumor-induced remodelling of genetically normal adjacent tissues offers opportunities for the development of novel anti-cancer strategies. A key mediator of interactions between tumor cells and their surroundings is extracellular matrix (ECM). For example, stiffening of interstitial ECM in the vicinity of breast tumors promotes metastasis [1]. One mechanism behind this observation appears to be cross-linking of collagen and elastin fibers by enzymes of the lysyl oxidase (LOX) family in the cancer-associated stroma, which provides an enhanced substrate for focal adhesion formation and pro-migratory signaling in tumor cells [2]. Aberrant expression of laminin, another key ECM component, is also observed in many cancers and may contribute to regulation of cancer stem cells, cell invasion, angiogenesis, and drug resistance [3, 4].

A major bottleneck in counteracting tumor-driven microenvironment remodeling is the dearth of tools to combat pathological forms of ECM assembly or modification. Enzymes provide powerful control points in biological processes, since the effects of their inhibition or augmentation are amplified by catalytic turnover. In addition to the LOX family, other cross-linking enzymes affect the physical and functional properties of the ECM and contribute to the tumor microenvironment. In particular, disulfide bond formation in the ECM of fibroblast cells is required for assembling a matrix capable of supporting tumor cell adhesion and migration [5]. The catalyst in this context is Quiescin sulfhydryl oxidase 1 (QSOX1), an enzyme that is found in the Golgi apparatus of most cell types but is up-regulated and secreted from fibroblasts actively producing ECM precursors [6]. QSOX1 transcripts were found at higher levels in the stroma of aggressive breast carcinomas [7], and QSOX1 expression is up-regulated in a variety of adenocarcinomas including breast, lung, pancreas, and prostate [8–12]. Motivated by these findings, we generated monoclonal antibody inhibitors of QSOX1 that function by sterically blocking the enzyme active site [13, 14]. We observed that inhibition of QSOX1 during fibroblast growth prevented formation of the copious pro-migratory ECM deposited by these cells, resulting in a failure of tumor cells to penetrate the fibroblast layer [5]. This finding identified a novel point for intervention in stromal support of tumors and established QSOX1 inhibitory antibodies as a means to affect this process. QSOX1 modifies ECM extracellularly [5], so excess QSOX1 produced by cancer-associated stromal fibroblasts in a physiological context is expected to be accessible for blocking by antibodies administered systemically.

This report is the first to address whether inhibition of secreted QSOX1 affects tumor progression in cancer models *in vivo*. To initiate this study, mice bearing syngeneic 4T1 tumors were treated with antibody specific for murine QSOX1. Tests were then extended to a melanoma model and to a human breast cancer xenograft. The latter was treated with an antibody combination blocking QSOX1 from both murine and human sources. Consistent findings were observed in multiple independent experiments, and an investigation of the mechanistic basis for QSOX1 inhibitory antibody *in vivo* extends previous observations made using cell culture mimetics of tumor-stromal interactions.

## RESULTS

### QSOX1 expression and secretion are induced in tumor-associated stromal cells

**Figure 1:**
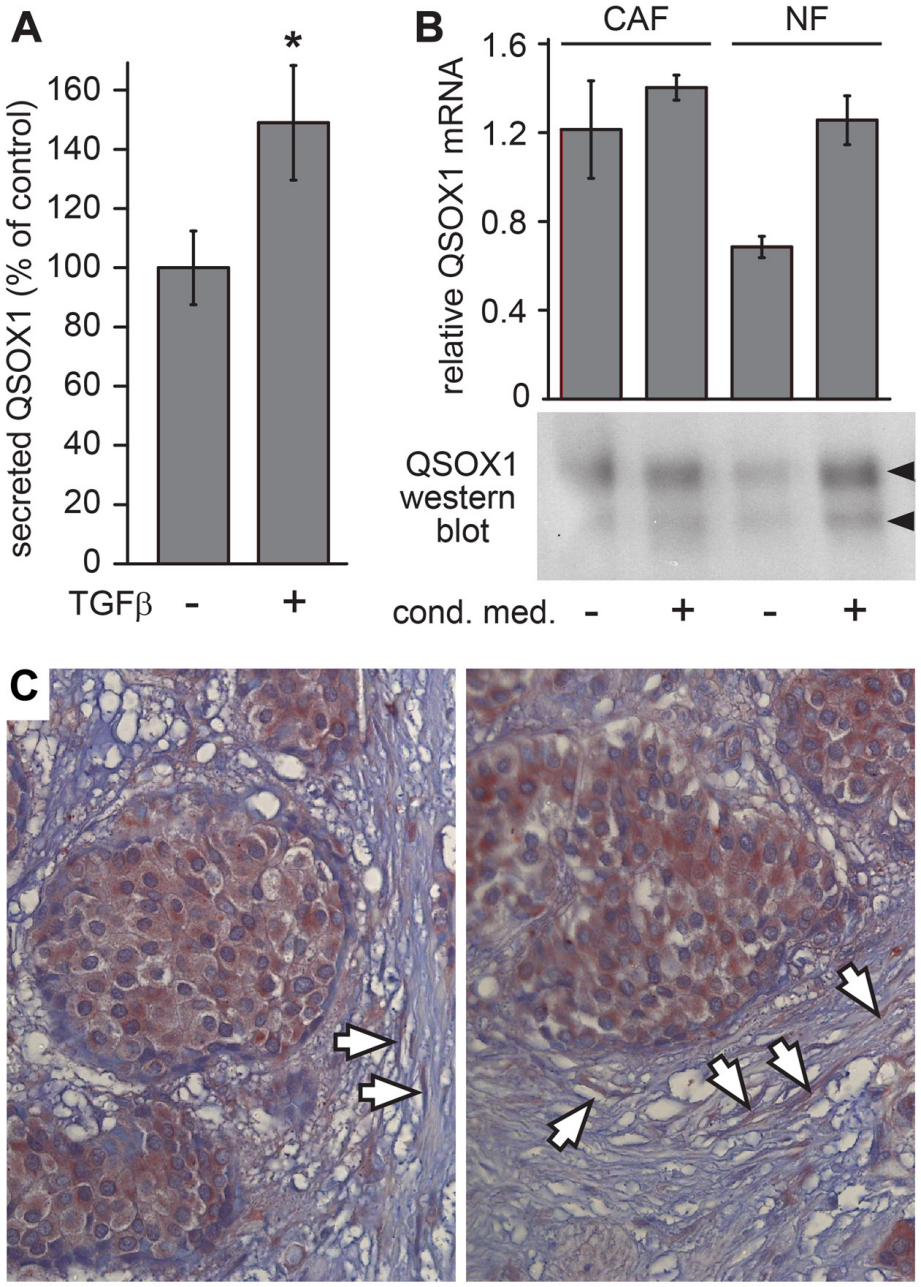
QSOX1 production by tumor-associated fibroblasts. (**A**) Parallel cultures of sub-confluent WI-38 fibroblasts were either treated with TGF-β (+) or left untreated (–), and the amount of QSOX1 in the medium after 48 hours was quantified by western blot. Error bars are standard error from four biological replicates (*p*-value < 0.01). (**B**) Primary fibroblast cultures derived from biopsy of a lung cancer patient were evaluated for QSOX1 transcript levels and secreted QSOX1 protein. CAF and NF cultures were grown with or without conditioned medium from a tumor cell line prior to mRNA quantification and protein analysis by western blot. The two QSOX1 bands, indicated by arrowheads, arise from the two QSOX1 splice variants, as observed previously [5]. Error bars are standard deviation from three parallel cultures. (**C**) A breast tumor biopsy was IHC stained for QSOX1. Intense QSOX1 staining is seen within the tumors, and arrows indicate highly stained fibroblasts in the tumor vicinity.

Treatment of non-quiescent fibroblasts with TGF-β, a key regulator of tumor microenvironment signaling pathways [15] and a driver of fibrotic ECM deposition [16], was previously shown to induce QSOX1 transcription [6]. To determine whether QSOX1 may be a factor by which TGF-β influences the extracellular environment, we tested whether TGF-β also upregulates QSOX1 on the protein level. Addition of TGF-β resulted in increased QSOX1 secretion from pre-confluent primary fibroblasts compared to parallel control cultures (Figure 1A).

The finding that tumor signaling factors can modulate the levels of extracellular QSOX1 led to the hypothesis that cancer-associated fibroblasts (CAFs) may secrete higher levels of QSOX1. To test this hypothesis, we measured QSOX1 secretion *ex vivo* by CAFs and by control fibroblasts (conventionally referred to as normal fibroblasts; NFs) from the same lung cancer patient but remote from the tumor. CAFs showed higher QSOX1 transcription and secreted protein levels than NFs (Figure 1B). However, supplementing primary NF cultures with conditioned medium from H460 human lung cancer cells, which do not secrete detectable levels of QSOX1 [5], increased QSOX1 expression to a comparable level as seen in CAFs (Figure 1B). These results show that increased QSOX1 secretion is a feature of human CAFs.

To analyze QSOX1 expression in tumor stroma *in situ*, we performed immunohistochemical (IHC) staining on paraffin sections of biopsies from breast cancer patients. QSOX1 staining was pronounced in fibroblasts adjacent to tumors (Figure 1C). As QSOX1 secreted from fibroblasts is likely to have been washed from the tissue sections during sample processing, this staining may not represent the totality of QSOX1 enzyme in the tumor vicinity. Nevertheless, the findings in CAF cultures and tumor sections together demonstrate increased production and secretion of QSOX1 by fibroblasts associated with tumors.

### QSOX1 inhibitory antibody decreased tumor growth in a syngeneic breast cancer model

**Figure 2:**
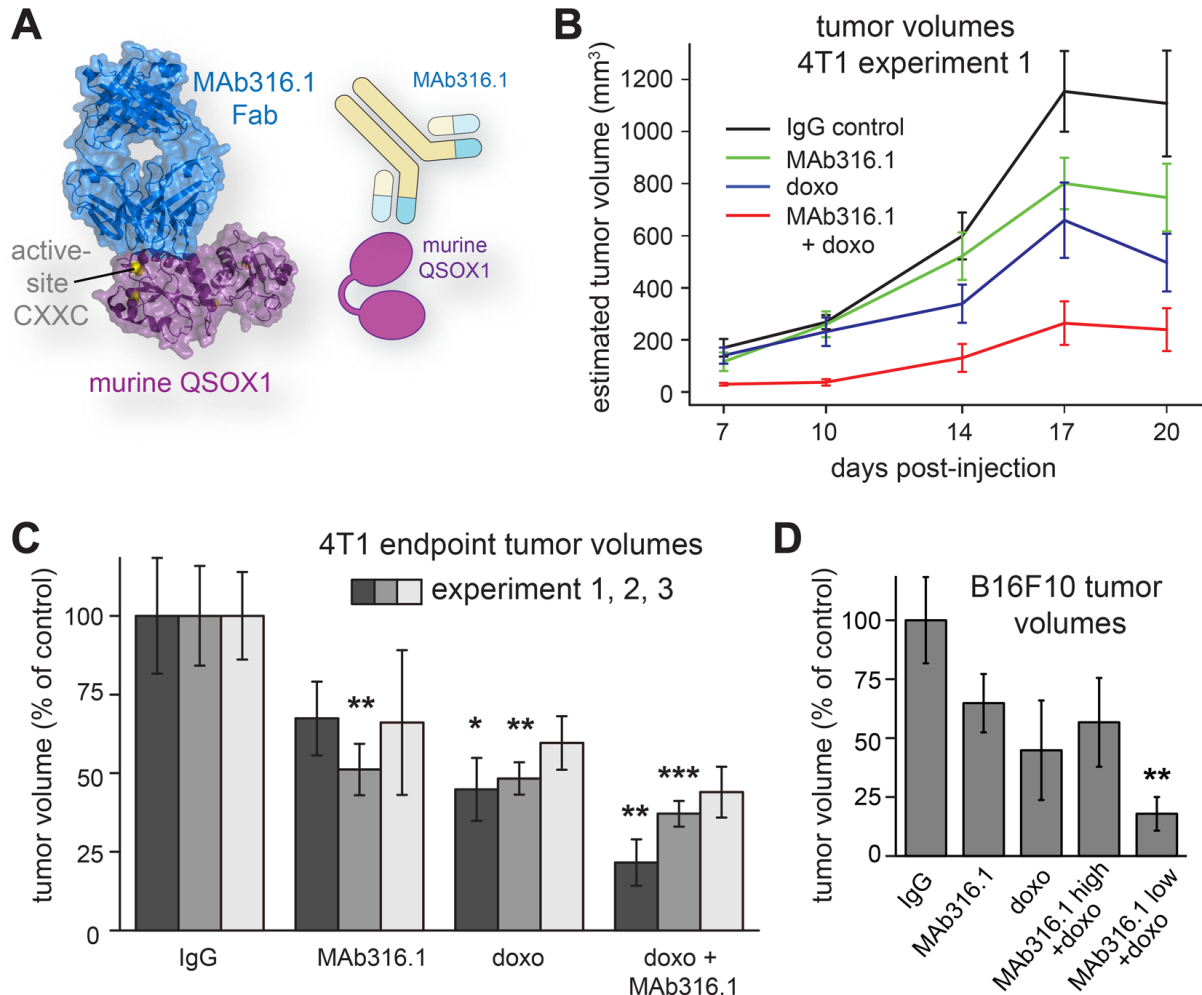
Syngeneic mammary tumor and melanoma models treated with QSOX1 inhibitory monoclonal antibody and chemotherapy. (**A**) MAb316.1 inhibits murine QSOX1. Image is based on protein data bank entry 5D93. (**B**) Mice bearing 4T1 tumors were treated with control IgG, MAb316.1 alone (30 mg/kg), doxorubicin, (8 mg/kg), or a combination of doxorubicin and MAb316.1. Reported tumor volumes were measured externally on the indicated days and averaged for each treatment group. (**C**) At the endpoints of three separate experiments (dark, medium, and light gray bars), tumors were removed and measured. (**D**) Mice bearing B16F10 melanoma tumors were treated with control IgG, MAb316.1 alone (50 mg/kg), doxorubicin (8 mg/kg), or a combination of doxorubicin and MAb316.1 (50 mg/kg or 25 mg/kg). Tumor volumes were measured at the experiment endpoint. Asterisks indicate *p*-values compared to IgG control (^*^ < 0.05; ^**^ < 0.01; ^***^ < 0.001).

We previously observed that inhibition of QSOX1 in fibroblasts *in vitro* prevented the adhesion and migration of co-cultured tumor cells [5]. To test the effect of QSOX1 inhibition on tumor progression *in vivo*, we used the mouse 4T1 syngeneic triple negative breast cancer (TNBC) model. Cultured 4T1 tumor cells were injected into mouse mammary fat pads. Treatment regimens began after tumor growth was visually validated. Mice were treated either with inhibitory monoclonal antibody specific for murine QSOX1 (MAb316.1) [14] (Figure 2A), with the widely-used chemotherapeutic reagent doxorubicin, or with a combination of both. Tumor volumes estimated from external measurements during the course of the experiment were lower following treatment with MAb316.1 than with an IgG control antibody (Figure 2B). As expected, doxorubicin treatment resulted in smaller tumor volumes as compared with control, and the combination of doxorubicin and MAb316.1 resulted in even lower tumor volumes (Figure 2B). At the experiment endpoint, tumor volumes were measured *ex vivo*, recapitulating the volume estimates during the course of treatment (Figure 2C). In summary, tumor volumes of the group receiving doxorubicin and MAb316.1 remained lower not only than the control group but also than the groups receiving chemotherapy or MAb316.1 alone (Figure 2C).

To establish reproducibility and enable further analyses of the tumors, two additional, independent experiments using the 4T1 model were performed. In both, tumor volumes at the experiment endpoint were lowest for the group receiving a combination of doxorubicin and MAb316.1 (Figure 2C). In addition, mice treated with MAb316.1 alone had reproducibly lower tumor volumes than the control group, comparable with the doxorubicin-treated group (Figure 2C). No effect on cell proliferation was observed when the MAb316.1 antibody was added to 4T1 cells in culture (Supplementary Figure 1). Tumor growth inhibition by the antibody observed *in vivo* is thus consistent with participation of the tumor microenvironment, as supported by further experiments described below.

### QSOX1 inhibition decreased tumor growth in a syngeneic melanoma model

We next tested whether the effect of QSOX1 inhibition on tumor growth is applicable to other cancer types. B16F10 melanoma is another well established and widely used murine model for the study of tumor growth and lung metastasis [17]. B16F10 cells were injected subcutaneously into syngeneic mice. After tumor growth was visually validated, mice were treated with MAb316.1, doxorubicin, or combinations of both. As B16F10 cells grew faster than 4T1 cells *in vitro*, two antibody concentrations were used, one dose (25 mg/kg) similar to the 4T1 model, and a higher dose (50 mg/kg). Tumor growth rates were more variable in this model compared to 4T1, but average tumor volumes at endpoint showed similar trends as observed in the breast cancer model. Specifically, treatment with MAb316.1 alone led to smaller average tumor size compared to treatment with IgG control, and a combination of doxorubicin and MAb316.1 resulted in the lowest tumor volumes (Figure 2D).

### Decreased metastasis following QSOX1 inhibitory antibody treatment

**Figure 3:**
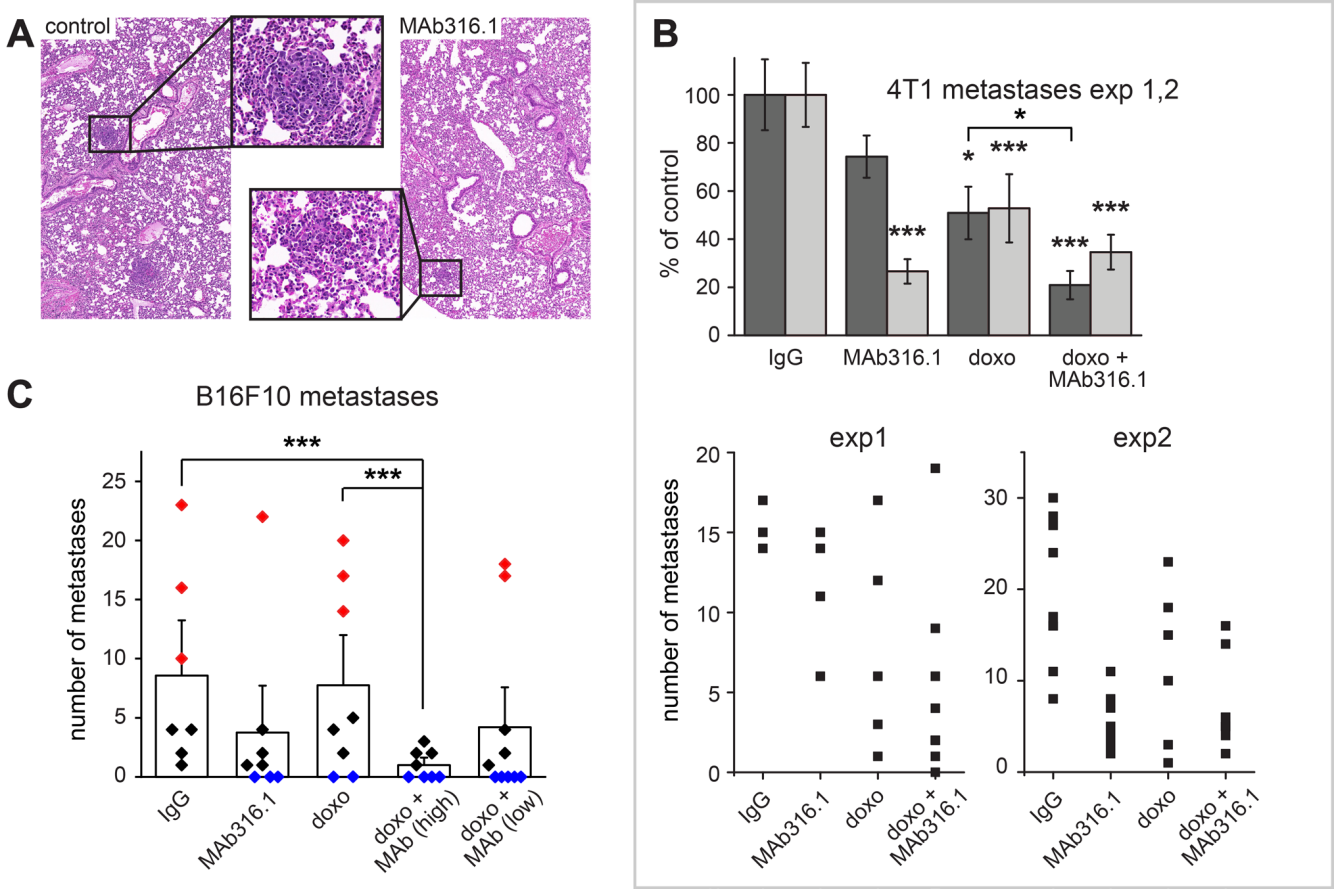
Lung metastases in 4T1 and B16F10 models. (**A**) Representative images of H&E stained lung sections from a 4T1 experiment, taken at 45× magnification. One metastasis from each section is boxed and shown in detail. (**B**) Number of lung metastases in treatment groups from two experiments with the 4T1 model. Top panel, averages for each treatment group as percent of IgG control. Asterisks indicate *p*-values compared to control (^*^ < 0.05; ^***^ < 0.001). Bottom panels, numbers of metastases in individual mice. (**C**) Lung metastases in B16F10 model. Bars denote the average number of metastases for each treatment. Rhombi represent the number of metastases in individual mice (red, ten or more metastases; blue, no metastases). Asterisks indicate *p*-values (^*^ < 0.05).

4T1 cells are highly metastatic and show spontaneous migration to the lungs [18]. Based on our finding that secreted QSOX1 contributes to tumor cell migration in cell cultures [5], we examined the effect of extracellular QSOX1 inhibition on metastasis. At the endpoints of two independent 4T1 experiments, lungs were fixed, embedded in paraffin, and metastases were counted (Figure 3A). The number of metastases generally correlated with tumor size, such that fewer metastases were observed in mice treated with MAb316.1 compared to mice receiving IgG control antibody. Furthermore, treatment with MAb316.1 combined with doxorubicin resulted in fewer metastases compared to treatment with doxorubicin alone (Figure 3B). A similar decrease in metastasis was seen in the B16F10 model. In the B16F10 groups treated with IgG control antibody or doxorubicin, a wide spread in the number of metastases was observed, with six out of 15 mice showing more than ten metastases in the lung sections examined (Figure 3C). In contrast, across all groups receiving MAb316.1, only three out of 26 mice had ten or more metastases. Furthermore, many more metastasis-free lungs were found in antibody-treated mice (twelve of 26) compared to the set of mice that received IgG control or doxorubicin (two of 15, all in the doxorubicin group) (Figure 3C).

### QSOX1 inhibitory antibodies impaired tumor growth in a xenograft breast cancer model

**Figure 4:**
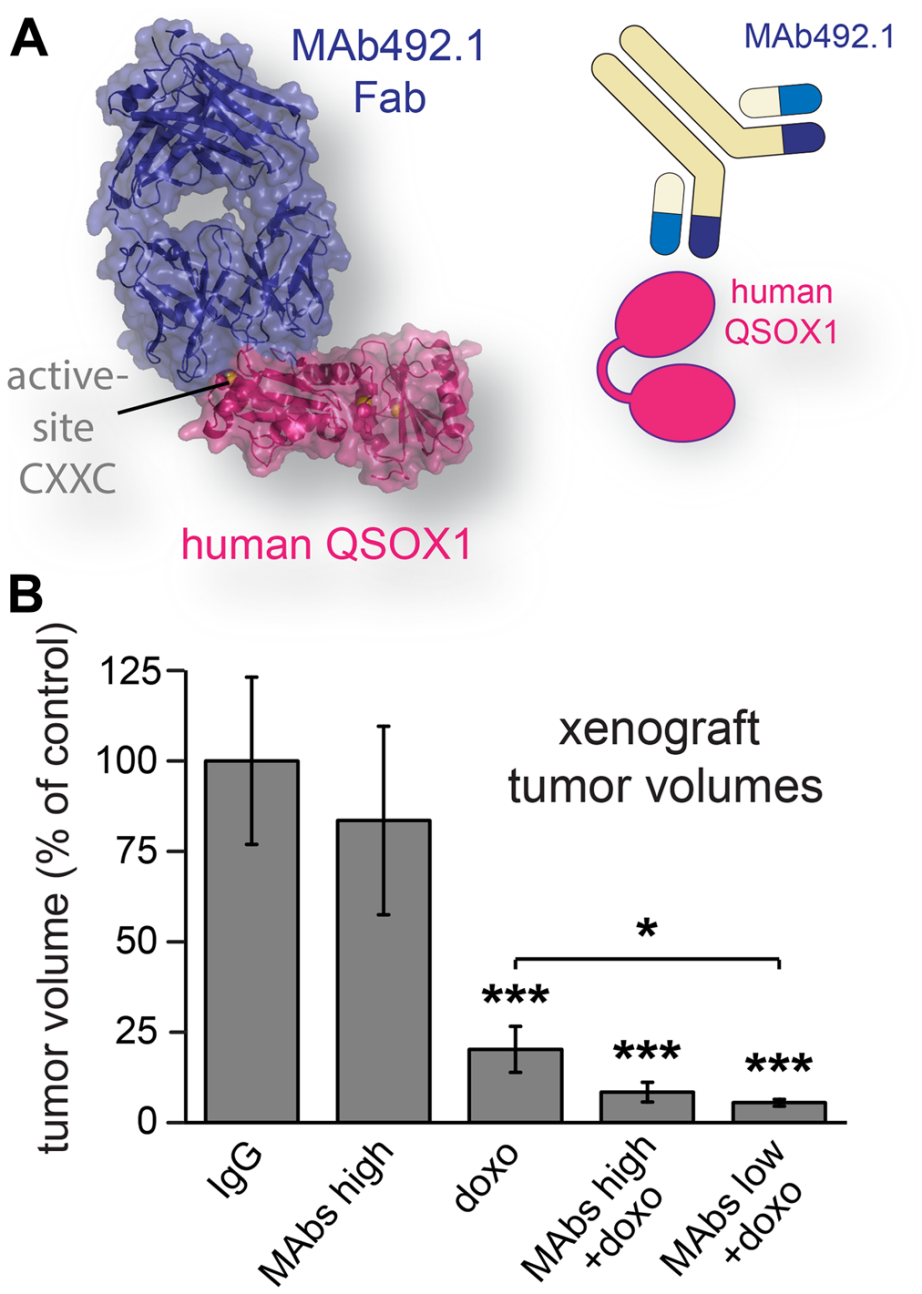
Human breast cancer xenograft model treated with QSOX1 inhibitory monoclonal antibodies and chemotherapy. (**A**) MAb492.1 inhibits human QSOX1. (**B**) Mice were treated with control IgG, a combination of MAb316.1 and MAb492.1 (30 mg/kg, and 25 mg/kg, respectively), doxorubicin (8 mg/kg), or combinations of doxorubicin, MAb316.1 (30 mg/kg) and MAb492.1 (25 mg/kg or 10 mg/kg). Tumor volumes were measured at endpoint. Asterisks indicate *p*-values (^*^ < 0.05; ^***^ < 0.001).

We next examined the effect of inhibiting secreted QSOX1 in a xenograft model for human breast cancer. Cells of the aggressive basal human breast carcinoma line MDA-MB-231 [19] were injected into the mammary fat pads of nude mice, and treatment was started once tumor growth was established. In this model, antibody treatment consisted of a mixture of species-specific QSOX1 inhibitory antibodies [13, 14]. As for the syngeneic experiments described above, MAb316.1 was used to inhibit QSOX1 secreted from murine tissues, *i. e*., mouse stromal cells. Although no effect on cell proliferation was seen upon addition of the human QSOX1-specific inhibitory antibody MAb492.1 (Figure 4A) to MDA-MB-231 cultures (Supplementary Figure 1), MAb492.1 was added to the treatment regimen to block any QSOX1 that may have been secreted by MDA-MB-231 cells *in vivo*. The antibody combination was administered alone or with doxorubicin, and a group of mice was treated with doxorubicin only. As for the syngeneic models described above, tumor volumes in the xenograft group treated with antibodies and doxorubicin were smaller compared to both control and doxorubicin-treated groups (Figure 4B). MDA-MB-231 tumors grew rapidly and reached the maximum permissible size before metastases were observed. Additionally, severe necrotic collapse occurred in a fraction of the tumors in each group, and mice carrying these tumors were removed from the quantification, as described in the Materials and Methods. In the 4T1 model, in which necrosis was less severe, no significant difference in the fraction of necrotic tissue was detected in tumor sections following treatment with MAb316.1 or control antibody (Supplementary Figure 2), indicating that inhibition of tumor growth by QSOX1 inhibitory antibody is not mediated by induction of tissue necrosis. Despite the technical limitations in the MDA-MB-231 experiment, these xenograft data show that extracellular inhibition of QSOX1 may also be beneficial for controlling the growth of human cancer cells *in vivo*.

### Treatment with QSOX1 inhibitory antibodies partially mitigated side effects of chemotherapy

**Figure 5:**
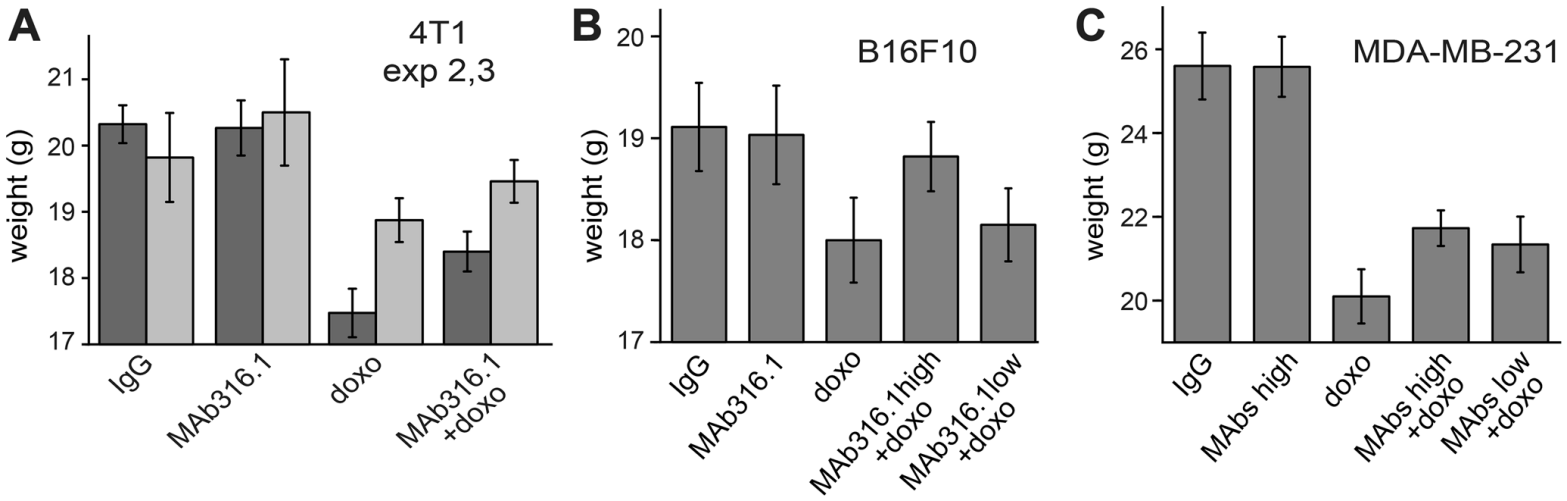
Body weights of mice following treatments with QSOX1 inhibitory antibodies and chemotherapy. Average body weights of mice at the endpoints of (**A**) the second and third 4T1 mammary tumor experiments, (**B**) the B16F10 melanoma experiment, and (**C**) the MDA-MB-231 breast tumor xenograft experiment. In the first 4T1 experiment, average weight loss upon doxorubicin treatment was less than 4% (data not shown), so an alleviating effect could not be measured.

During the course of this study, an unexpected effect of QSOX1 inhibitory antibody administration was observed. It is well known that chemotherapies in general, and doxorubicin in particular, have severe side effects, leading to weight loss and weakness in human patients. Similar effects are seen in mice receiving doxorubicin [20] and were observed in most of the experiments described above, as quantified by body weight (Figure 5). Remarkably, the body weights of mice receiving doxorubicin together with QSOX1 inhibitory antibodies were consistently higher at the experiment endpoint than the weights of mice receiving doxorubicin alone (Figure 5), and their general well-being appeared less compromised. The alleviating influence of QSOX1 antibodies on doxorubicin toxicity was detected in four out of five experiments and in all three tumor models. Administration of QSOX1 antibody alone did not have a noticeable effect on weight or any obvious side effects.

To test the generality of the impact of QSOX1 inhibitory antibody on side effects of chemotherapy, we administered either doxorubicin alone or doxorubicin in combination with MAb316.1 to mice not bearing tumors. As observed in the tumor treatment experiments, all animals lost weight due to doxorubicin administration. However, following two weeks of treatment, weight loss of mice receiving doxorubicin together with antibody was less severe than weight loss of mice receiving doxorubicin alone (Supplementary Table 1). Moreover, mice in the group treated with doxorubicin alone showed weakness and lack of well-being, requiring termination of the experiment, while mice receiving doxorubicin with antibody behaved normally. The observed effect of QSOX1 antibody is unlikely to be a general property of antibody administration together with doxorubicin, as similar experiments involving co-treatment with antibodies and doxorubicin were not documented as having a beneficial effect on weight loss or well-being (*e.g*., [21]). The mechanistic basis for the apparent attenuation of doxorubicin toxicity by QSOX1 inhibitory antibody is unknown, but a potential clinical benefit would be substantial.

### QSOX1 inhibitory antibodies alter the tumor microenvironment

**Figure 6:**
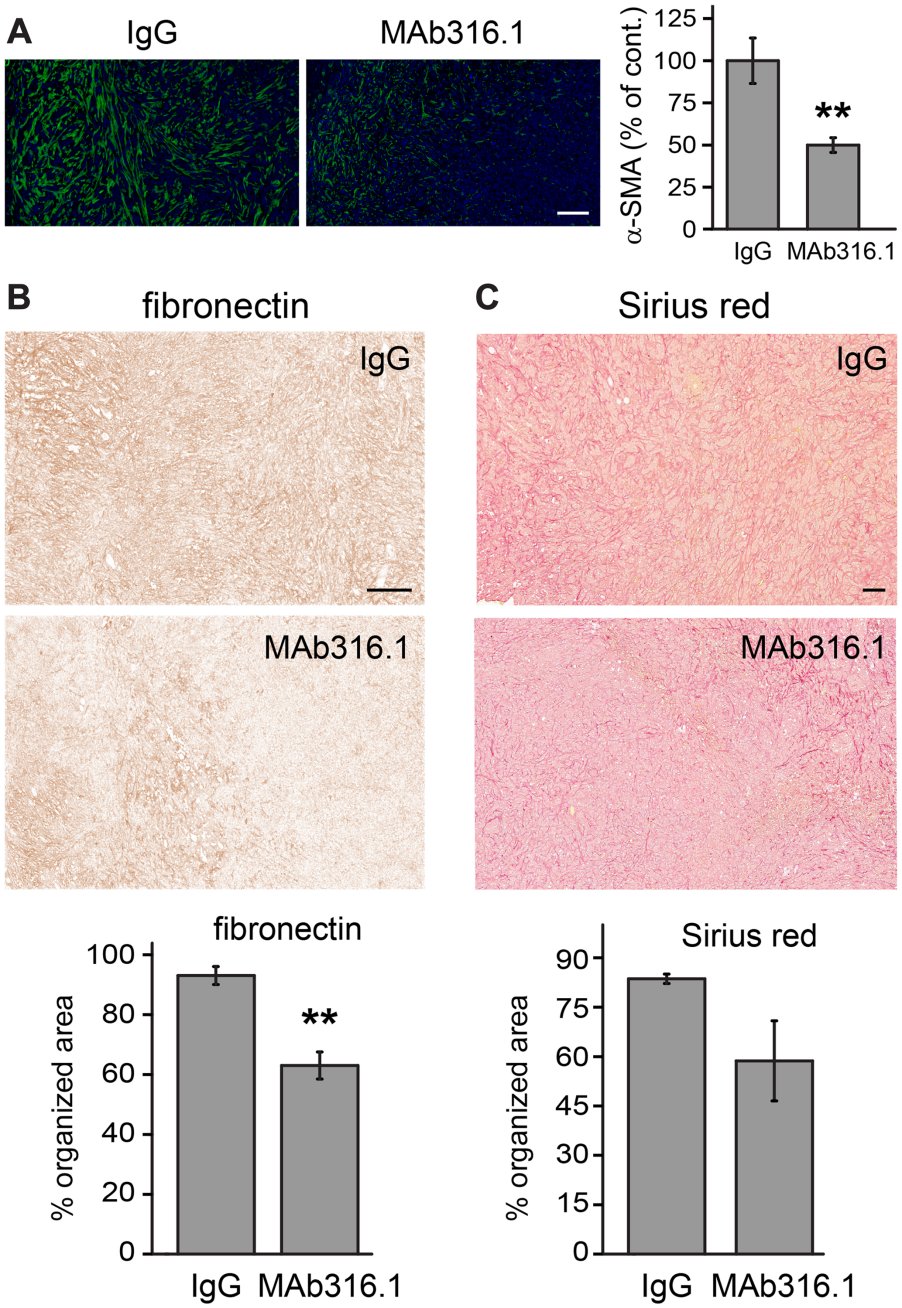
4T1 tumors from mice treated with MAb316.1 show decreased stromal participation. (**A**) Left, representative immunofluorescence images of tumor sections labeled for α-SMA and stained with DAPI. Scale bar is 100 μm. Right, quantification of α-SMA staining. (**B**) Top, representative images showing the organization of fibronectin in tumors as visualized by IHC staining. Scale bar is 200 µm. Bottom, percent of organized, filamentous area for fibronectin. (**C**) Top, representative images of Sirius red staining of tumors, indicating collagen. Scale bar is 200 µm. Bottom, percent of organized, filamentous area for Sirius red. Error bars are standard error. Asterisks indicate *p*-values compared to IgG control (^**^ < 0.01).

To gain insight into the mechanism by which QSOX1 inhibitory antibodies interfere with tumor progression *in vivo*, we examined the microenvironment of 4T1 tumors from control and antibody-treated mice. Tumors from animals that did not receive doxorubicin were chosen for this and the following experiments to focus on the effect of the antibody alone. A major characteristic of activated tumor microenvironment is the acquisition of smooth muscle features, especially formation of stress fibers and expression of α-SMA from differentiated myofibroblasts [22]. Decreased α-SMA staining was observed in tumors from MAb316.1-treated animals compared to control tumors (Figure 6A).

Previous *in vitro* observations showed differences in laminin incorporation into the ECM [5] and defects in organization of fibronectin [23] upon depletion or inhibition of QSOX1. High variability in laminin labeling of the 4T1 tumor sections compromised conclusive analysis (data not shown). However, two additional major ECM components, fibronectin and collagen, showed consistent differences between the control and antibody-treated groups. Whereas tumors from the control group showed extensive, well-organized networks of fibronectin and collagen, such networks were less evident or truncated in the MAb316.1 treatment group (Figure 6B, 6C and Supplementary Figure 3). The observed decrease in myofibroblasts and in ECM network organization support the conclusion that QSOX1 inhibition affects ECM in the tumor microenvironment.

We next evaluated whether QSOX1 inhibitory antibody treatment affected immune cell infiltration into 4T1 tumors. Substantially lower numbers of leukocytes (CD45+) were detected from the MAb316.1-treated tumors (Supplementary Figure 4). Further analysis revealed that the CD45+ fractions from all animals in the MAb316.1-treated group contained substantially more cell debris and aggregates than the CD45+ fractions from the control group (Supplementary Figure 4). It is possible that the tissue dissociation procedure affected the control and treated samples differently, consistent with the apparent differences in ECM organization described above. At this point we cannot rule out that differences in tumor mechanical properties led to an inability to detect leukocytes in the MAb316.1-treated mice. Alternatively, the lower numbers of leukocytes detected may have resulted from actual decreased leukocyte penetration through blood vessels in the MAb316.1-treated mice, perhaps due to altered ECM integrity.

## DISCUSSION

In this report we demonstrate the efficacy of inhibitory antibodies against the catalyst of disulfide bond formation QSOX1 in attenuating tumor growth and metastasis in cancer models in mice. QSOX1 is expressed at high levels in a variety of human adenocarcinomas [8–12] and is also over-produced by stromal fibroblasts associated with aggressive breast carcinomas [7]. It was previously observed that TGF-β induces transcription of QSOX1 by fibroblasts [6], and here we show that TGF-β stimulation also leads to increased QSOX1 secretion (Figure 1A). Furthermore, we observed that cultured lung CAFs expressed and secreted high levels of QSOX1 (Figure 1B). Excess QSOX1 secreted by CAFs is expected to be the target of inhibition relevant to the effects observed in this study. Our approach may be compared with the observation that treatment with Ebselen, a synthetic organoselenium compound reported to bind and suppress QSOX1 activity *in vitro*, attenuated tumor growth in a pancreatic xenograft model [24]. While targeting diverse Ebselen-sensitive thiol species *in vivo* may decrease tumor cell proliferation, the observed effects of treatment with this compound are not likely to be specific [25]. Focusing on the contribution of extracellular QSOX1 to the tumor microenvironment by specifically targeting the secreted enzyme is an alternative method to counter the participation of QSOX1 in adenocarcinomas.

It has been argued that modulating the synthesis or post-translational modification of specific ECM components is a promising approach to combating cancer [26]. Along these lines, our analysis of 4T1 tumors *ex vivo* revealed differences in ECM organization in the tumor microenvironment of animals treated with the QSOX1 inhibitory antibodies (Figure 6). Fewer myofibroblasts were found, as indicated by α-SMA staining, compared to tumors from animals treated with control IgG (Figure 6A). Furthermore, fibronectin and collagen networks in tumors from antibody-treated mice were less organized and interconnected than the networks of control animals (Figure 6B, 6C). These observations are consistent with our earlier findings on cultured fibroblasts grown without active QSOX1: mechanical stiffness of the ECM in these cultures was impaired, fibronectin matrix was perturbed, adhesion of tumor epithelial cells to the fibroblasts was compromised, and tumor cell migration through the fibroblasts and associated ECM was inhibited [5, 13, 23]. Since a growing tumor relies on supportive ECM structures for cell proliferation and dissemination of metastases, QSOX1 may be involved in a tumor-driven program to create a microenvironment favorable for cancer progression and is therefore an emerging target for therapeutic inhibition. Other approaches designed to directly ablate ECM-producing cells in the tumor vicinity have been shown to be counterproductive [27]. In contrast, QSOX1 inhibition has a more specific effect on tumor-associated fibroblasts and may alter the quality and properties of the matrix without destroying protective functions of the tumor stroma.

Combining agents affecting tumor stroma with other therapeutic approaches including immunotherapy [28] is an emerging strategy for enhancing treatment efficacy. Interfering with tumor-induced ECM remodeling by QSOX1 inhibition was envisioned as a supplementary therapy that would be combined with other anti-cancer treatments. The experiments described here were designed to monitor the contribution of QSOX1 inhibitory antibodies to a regime of standard chemotherapy, in addition to documenting their effect when administered alone. Smaller tumors and fewer metastases were found following treatment with QSOX1 inhibitory antibody than with control antibody. Furthermore, administration of QSOX1 inhibitory antibody in combination with doxorubicin provided an additional advantage over doxorubicin or antibody alone. Given that the two treatments were efficacious individually, the higher potency of the treatment combination is consistent with the expectation that QSOX1 inhibition and doxorubicin affect different aspects of tumor growth and may therefore function in a complementary manner. Doxorubicin is directly cytotoxic, whereas QSOX1 inhibition undermines the contribution of the ECM to tumor development. Defects in microenvironment and ECM organization upon QSOX1 inhibition *in vivo* could compromise the physical and signaling support required by tumor cells, leading to the observed decreased tumor growth and metastasis.

Treatments targeting the tumor microenvironment may be particularly important in TNBC, which comprises 10–20% of breast cancer cases and has high rates of metastasis. TNBC patients have lower survival rates compared to patients with other breast cancer subtypes [29] and are not treatable by the targeted therapies available for other breast cancer subtypes. Currently, TNBC patients undergo rigorous but generic therapies consisting of surgery, chemotherapy, and radiotherapy [30]. There is room for development of additional therapeutics to complement or increase the efficacy of these conventional treatments. Of particular value would be treatments specifically targeting the support system provided by the tumor-associated stroma without eliciting additional severe side effects.

The 4T1 murine mammary carcinoma is a TNBC commonly used as an experimental animal model for human breast cancer [31]. Enhanced QSOX1 transcription was found in 4T1 cells removed from lung metastases, compared to cells from the main tumor [32], suggesting that QSOX1 may be involved in the dissemination and infiltration of 4T1 tumors. Though it is not yet known how stromal fibroblasts respond to the proliferation of 4T1 cells in their vicinity, the cumulative observations, presented here (Figure 1) and elsewhere [7], that QSOX1 secretion is induced in CAFs suggested that 4T1 tumors may be responsive to treatment with QSOX1 inhibitors. Indeed, in a series of independent experiments, we found that to be the case. Beneficial effects of QSOX1 inhibitory antibodies were reproducible in three 4T1 experiments, when administered alone or in combination with doxorubicin.

The efficacy of QSOX1 inhibitors was not restricted, however, to the syngeneic 4T1 model. Similar effects as in the repeated 4T1 study were observed in a xenograft involving human breast cancer-derived cells, as well as in an aggressive mouse melanoma model. These various models were conducted using the appropriate mouse strain in each case. The properties of the tumor cells and the genetic backgrounds of the mice were thus different in the different experiments. The reproducibility and relevance of QSOX1 inhibition to multiple cancers in various mouse strains strengthens the notion that stromal support is a general requirement for diverse cancer types and hosts. As the stroma lacks the dynamic genetic changes occurring in epithelial tumors, it is expected to be a more stable target for therapeutic modulation.

A more surprising observation made in the 4T1 model and in other experiments conducted in this study is that mice receiving a combination of doxorubicin and QSOX1 inhibitory antibody demonstrated better well-being than animals treated with doxorubicin alone. Animals receiving both treatments had higher body weights (Figure 5) and increased locomotion (unpublished observations) than animals receiving only chemotherapy. Although the mechanism underlying this effect is not known, it is an important observation, as side effects of chemotherapy often dictate the dosage and duration of treatment [33, 34]. Possible explanations for the partial amelioration of chemotherapy side effects by the QSOX1 inhibitory antibody can be considered. Attenuating the potency of the chemotherapy would be one mechanism for decreasing side effects, but addition of doxorubicin to the QSOX1 inhibitory antibody treatment regimen was clearly beneficial therapeutically, indicating that the chemotherapy was still potent in the presence of the antibody. Since QSOX1 is found in the circulation [35], as well as in organs of the gastrointestinal tract, both of which contain cell populations that proliferate rapidly, QSOX1 inhibition may affect how these organs respond to chemotherapy.

Antibodies are increasingly used in cancer treatment cocktails and for other therapeutic purposes owing to their high specificity and biocompatibility. QSOX1 inhibitory antibodies have been well-characterized and show high affinity to their target [13, 14]. Our observation that QSOX1 inhibition with antibodies does not induce detectable side effects and also appears to lessen the severity of these side effects during the course of chemotherapy suggests that adding QSOX1 inhibitory antibodies to human cancer therapies may have merit in the treatment of TNBC and other cancers. To enable progress in this direction, the murine anti-human QSOX1 monoclonal antibody has been further developed as a chimera with human constant regions [36], toward assessment in clinical tests. In summary, we provide the first evidence that inhibition of QSOX1 with a specific inhibitory antibody affects tumor growth and metastasis *in vivo*.

## MATERIALS AND METHODS

### TGF-β induction of QSOX1 expression

Sub-confluent WI-38 fibroblast cultures were supplemented with 5 nM transforming growth factor beta (TGF-β) for 48 hr. Culture media were analyzed for QSOX1 secretion by western blot using QSOX1 polyclonal antibody [5].

### QSOX1 expression and secretion from CAFs and NFs

Cultured media and RNA samples from CAF and NF cells were obtained from the laboratory of Prof. Moshe Oren [37]. CAFs and NFs were isolated from a surgically resected lung tumor or from a grossly normal part of the same specimen, respectively. Conditioned medium was derived from a lung epithelial cell line (H460) grown in low serum (0.1%). QSOX1 secretion was determined for CAFs and NFs grown in either normal or conditioned medium, and was analyzed by western blot using QSOX1 polyclonal antibody. RNA was purified from cells and analyzed by RT-PCR using the following primers: QSOX1: forward 5′-GAAATTGGCAGATCGCTCCA-3′, reverse 5′-GCCCACTTCTATCCGCAGG-3′, and GAPDH: forward 5′- ACCCACTCCTCCACCTTTGA-3′, reverse 5′- TGTTGCTGTAGCCAAATTCGT-3′.

### IHC of human tumor sections

Paraffin sections from breast tumors (infiltrating duct carcinoma, grade II) were immunostained with QSOX1 polyclonal antibody according to a previously described protocol [38].

### Cell lines

4T1 cells were purchased from ATCC. MDA-MB-231 breast cancer cells were obtained from Prof. Yosef Yarden, Weizmann Institute of Science, and B16F10 melanoma cells were obtained from Prof. Gideon Schreiber, Weizmann Institute of Science. All cell lines were grown in DMEM culture medium supplemented with FCS (10%), L-glutamine (2 mM), and antibiotics. All cells were tested for mycoplasma prior to injection (EZ-PCR mycoplasma test kit, Biological Industries, Inc.).

### *In vivo* experiments

Animal experiments were approved by the Weizmann Institute Animal Care and Use Committee (IACUC) following U. S. National Institute of Health, European Commission, and Israeli guidelines. All mice were purchased from Envigo and randomly assigned to experimental groups.

### Syngeneic tumor models

Monoclonal inhibitory antibodies against human and murine QSOX1 were generated in our laboratory, as described [13, 14]. 4T1 breast cancer cells (250,000 in 200 μl 1:1 HBSS: Cultrex^®^) were injected orthotopically into mammary fat pads of 8-week old female BALB/c mice. B16F10 (50,000 in 100 μl 1:1 HBSS: Cultrex^®^) were injected subcutaneously into 8-week old female C57BL/6 mice. After injection, mice were randomized into treatment groups. Treatments were started 3 days after cell injection, once tumors were visible. Group size varied between experiments, 9 mice per treatment group in the first and second 4T1 experiments, 3–5 mice in the third 4T1 experiment, and 9–10 mice in the B16F10 model. Control animals received generic murine IgG injections (LifeSpan BioScience, Inc.), and experimental treatment groups received MAb316.1 alone (30 mg/kg in the 4T1 model, and 50 mg/kg in the B16F10 model), chemotherapy (doxorubicin, 8 mg/kg), or a combination of doxorubicin (8 mg/kg) and MAb316.1 (30 mg/kg in the 4T1 model, and 50 mg/kg or 25 mg/kg in the B16F10 model). Treatments were administered intraperitoneally (i. p.) in a maximum volume of 200 μl. Doxorubicin was administered once weekly and antibody twice weekly. Twice a week mice were weighed, and tumor size was externally measured using a caliper. Experiments were terminated 14 to 21 days after tumor cell injection, once the largest tumors reached the ethically permissible size or other signs of distress were observed. IACUC approval numbers for these experiments are: 25170216-1, 28380716-3, 04490618-2 for the 4T1 experiments, and 34190317-2 for the B16F10 model.

### Xenograft tumor model

Human breast adenocarcinoma MDA-MB-231 cells (200,000 in 200 ml 1:1 HBSS: Cultrex^®^) were orthotopically injected into mammary fat pads of 8-week old female nude mice. Following injection, mice were randomized into treatment groups with 7–8 mice per group. Treatments were started 3 days after cell injection, once tumors were visible. Similarly to the syngeneic models, mice received either control antibody, QSOX1-specific antibody, chemotherapy, or a combination of QSOX1-specific antibody and chemotherapy. However, as this model involves human-derived tumor cells engrafted into mice, both anti-human QSOX1 (MAb492.1, 25 mg/kg or 10 mg/kg) and anti-mouse QSOX1 (MAb316.1, 30 mg/kg) were administered to groups receiving QSOX1-specific antibodies. Treatment regimen was otherwise similar to the syngeneic models. The experiment endpoint was on day 28 after tumor cell injection. The tumors of a few mice in each of the treatment groups underwent a severe necrotic collapse during the course of the experiment, and these mice were therefore excluded from further analysis. Criteria for exclusion was a decrease of 75% in tumor volume between two measurements. IACUC approval number for this experiment is 30160916-2.

### Immunohistochemistry of 4T1 tumors

Tumors from the second 4T1 experiment were removed, fixed, and embedded in paraffin at endpoint. Sections were then deparaffinized and labeled as follows: alpha smooth muscle actin (α-SMA) was detected with specific antibody (A5228, Sigma Aldrich) followed by fluorescently labelled secondary antibody. The area labelled by the antibody divided by the DAPI stained area was calculated using ImageJ as an indication of myofibroblast differentiation. Fibronectin and laminin were detected using specific antibodies (A0245, Dako or 11575, Abcam, respectively) followed by 3,3′-diaminobenzidine (DAB) staining. Collagen was stained with Sirius red. Labeled areas were segmented using the WEKA Trainable Segmentation plugin to calculate the organized, filamentous fraction within each area. For each ECM protein, identical segmentation criteria were set for all scans. At least four slices from four representative tumors of IgG control and MAb316.1 treatment groups were analyzed for each experiment.

### Quantification of metastases and necrosis

Numbers of lung metastases were manually determined from hematoxylin and eosin (H&E) stained paraffin sections. Slides were scanned (Pannoramic SCAN, 3DHISTECH), and 3 sections, separated by 150 μm, were analyzed for each mouse. Necrotic tissue was measured from H&E stained tumor sections. Nine tumors, four slices from each, were stained and analyzed for each treatment group. Analyses were performed by two individuals in a double-blind fashion.

### FACS analysis of immune cells within tumors

Tumors from the third 4T1 experiment were removed at endpoint and immediately processed into single cell suspensions using the Tumor Dissociation Kit (Miltenyi Biotech) according to the manufacturer’s instructions. Cells were then labeled using CD45-VioGreen antibody (Miltenyi Biotech). Flow cytometry analysis was performed on a BD FACSAria Fusion instrument (BD Immunocytometry Systems), controlled by BD FACS Diva software v8.0.1 (BD Biosciences). Analysis was performed using FlowJo software v10.2 (Tree Star).

### Statistical analysis

Data for tumor size and mice weight showed normal distribution. Differences among the treatment groups were calculated by analysis of variance (ANOVA) using the software R (version 3.5.1). Data for metastasis numbers showed asymmetric distribution. Statistical analysis of these data was done using a generalized linear model with a Poisson distribution. All error bars in figures represent standard error, except for Figure 1B where standard deviation was calculated.

## SUPPLEMENTARY MATERIALS


