# Correction: Inhibition of fibroblast secreted QSOX1 perturbs extracellular matrix in the tumor microenvironment and decreases tumor growth and metastasis in murine cancer models

**DOI:** 10.18632/oncotarget.27764

**Published:** 2020-10-06

**Authors:** Tal Feldman, Iris Grossman-Haham, Yoav Elkis, Patrick Vilela, Neta Moskovits, Iris Barshack, Tomer M. Salame, Deborah Fass, Tal Ilani

**Affiliations:** ^1^ Department of Structural Biology, Weizmann Institute of Science, Rehovot 7610001, Israel; ^2^ Almog Diagnostic, Shoham 6081513, Israel; ^3^ Felsenstein Medical Research Center, Sackler Faculty of Medicine, Tel Aviv University, Tel Aviv 6997801, Israel; ^4^ Institute of Pathology, Sheba Medical Center Tel Hashomer, Sackler Faculty of Medicine, Tel Aviv University, Tel Aviv 6997801, Israel; ^5^ Life Sciences Core Facilities, Weizmann Institute of Science, Rehovot 7610001, Israel

**This article has been corrected:** The funding information has been updated. The correct listing is given below:

This work was supported by the European Research Council [grants number 310649 and 825076].

Original article: Oncotarget. 2020; 11:386–398. 386-398. https://doi.org/10.18632/oncotarget.27438

