# Supplementary material for: Inhibition of fibroblast secreted QSOX1 perturbs extracellular matrix in the tumor microenvironment and decreases tumor growth and metastasis in murine cancer models
Source: Oncotarget. 2020 Jan 28;11(4):386–98. doi: 10.18632/oncotarget.27438 (PMC6996906; doi:10.18632/oncotarget.27438)
Supplement: Supplementary file 1 [file oncotarget-11-386-s001.pdf]

Inhibition of fibroblast secreted QSOX1 perturbs extracellular matrix in the tumor microenvironment and decreases tumor growth and metastasis in murine cancer models

SUPPLEMENTARY MATERIALS

Supplementary Table 1: Weights of tumor-free mice receiving doxorubicin with or without QSOX1 inhibitory antibody MAb316.1

weight change (g) day 1 – day 15		weight change (g) day 11 – day 15	
**doxo**	**doxo + MAb316.1**	**doxo**	**doxo + MAb316.1**
–	2.5	–	–0.4
3.6	1.7	2.0	–0.4
–	3.0	–	0.1
4.0	4.4	2.0	1.0
3.9	1.1	2.3	–0.4
2.0	0.3	1.1	–1.8
–	4.5	–	1.7
2.3	2.5	0.7	0.0
0.8	1.5	0.3	–0.1
1.7	0.2	0.7	0.2
averages +/– SE		averages +/– SE	
2.6 +/– 0.5	2.2 +/– 0.5	1.3 +/– 0.3	–0.01 +/– 0.3

Values displayed are weight loss of mice from day 1 (prior to treatment) to day 15 (after two doxorubicin treatments), or from day 11 to day 15. Three animals treated with doxorubicin alone died on day 14 (indicated by "–") and were not counted in the results at experiment endpoint. Although the difference in average weight loss over the whole experimental period between the two groups was within error (dark gray), the qualitative difference between the groups in terms of viability and well-being was noticeable and substantial. Furthermore, weight loss in the group treated with antibody slowed or reversed during the last four days (light gray; *p*-value = 0.008).

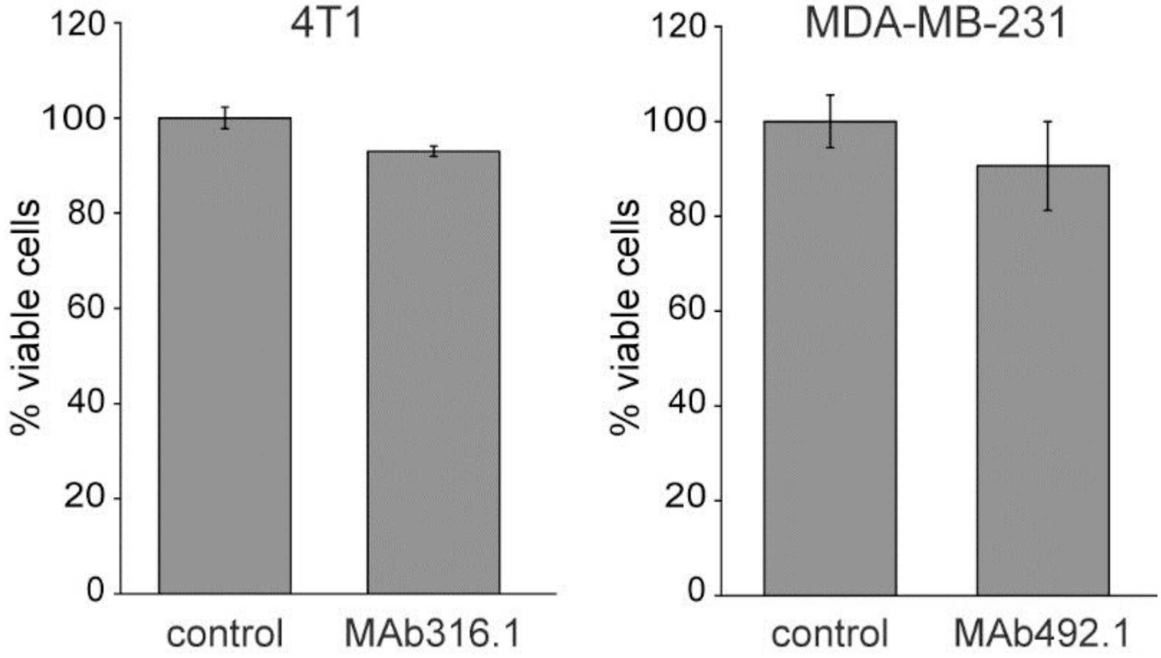

Supplementary Figure 1: Cultured cell proliferation in the presence of QSOX1 inhibitory antibodies. Numbers of viable 4T1 or MDA-MB-231 cells were determined 72 hours after the addition of 250 nM MAb316.1 or MAb492.1, respectively, to culture media as compared with control. Cells were fixed, DAPI stained, and imaged. Ten images were quantified per treatment group for each biological replicate ($n = 4$). Error bars represent standard error.

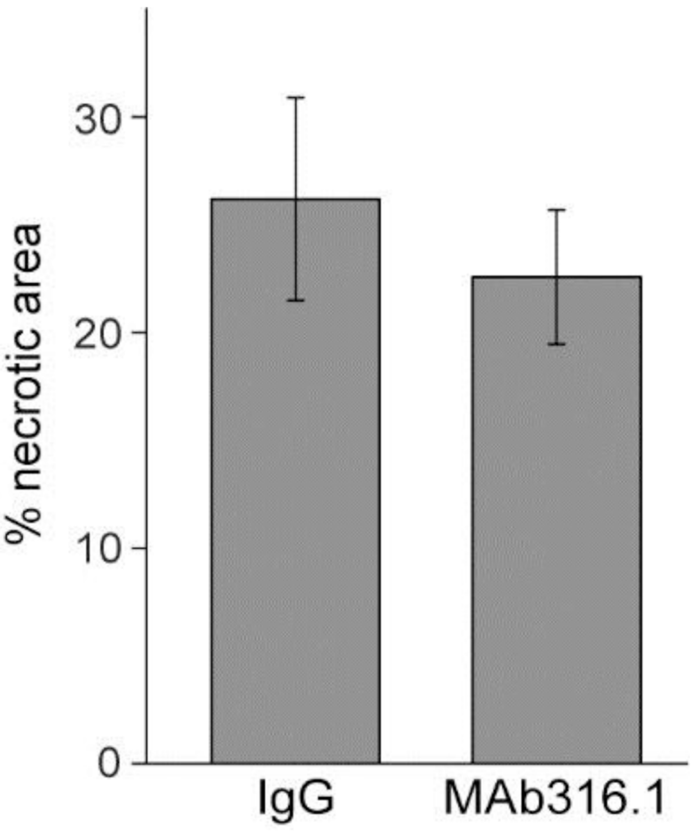

Supplementary Figure 2: Necrotic tumor tissue in the second 4T1 experiment. Percent necrotic area was determined for tumors from MAb316.1 and IgG control treatment groups. Four tumor sections from each mouse in this experiment were analyzed. Error bars represent standard error.

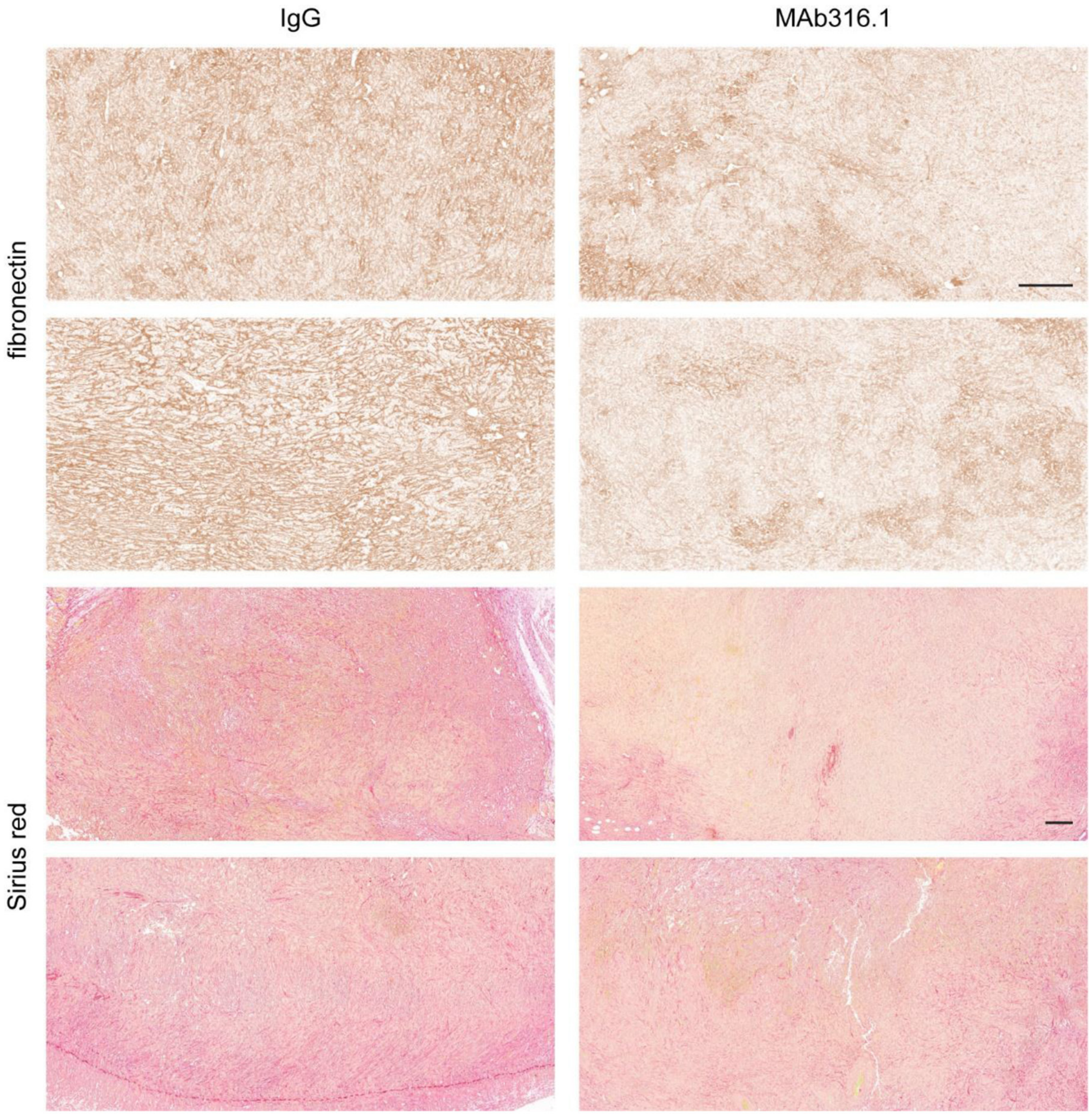

Supplementary Figure 3: Fibronectin and collagen staining in control and MAb316.1-treated tumors. Additional representative tumor sections as shown in Figure 6B and 6C, each taken from a different mouse, immunostained for fibronectin or labeled with Sirius red. Scale bars are 200 µm.

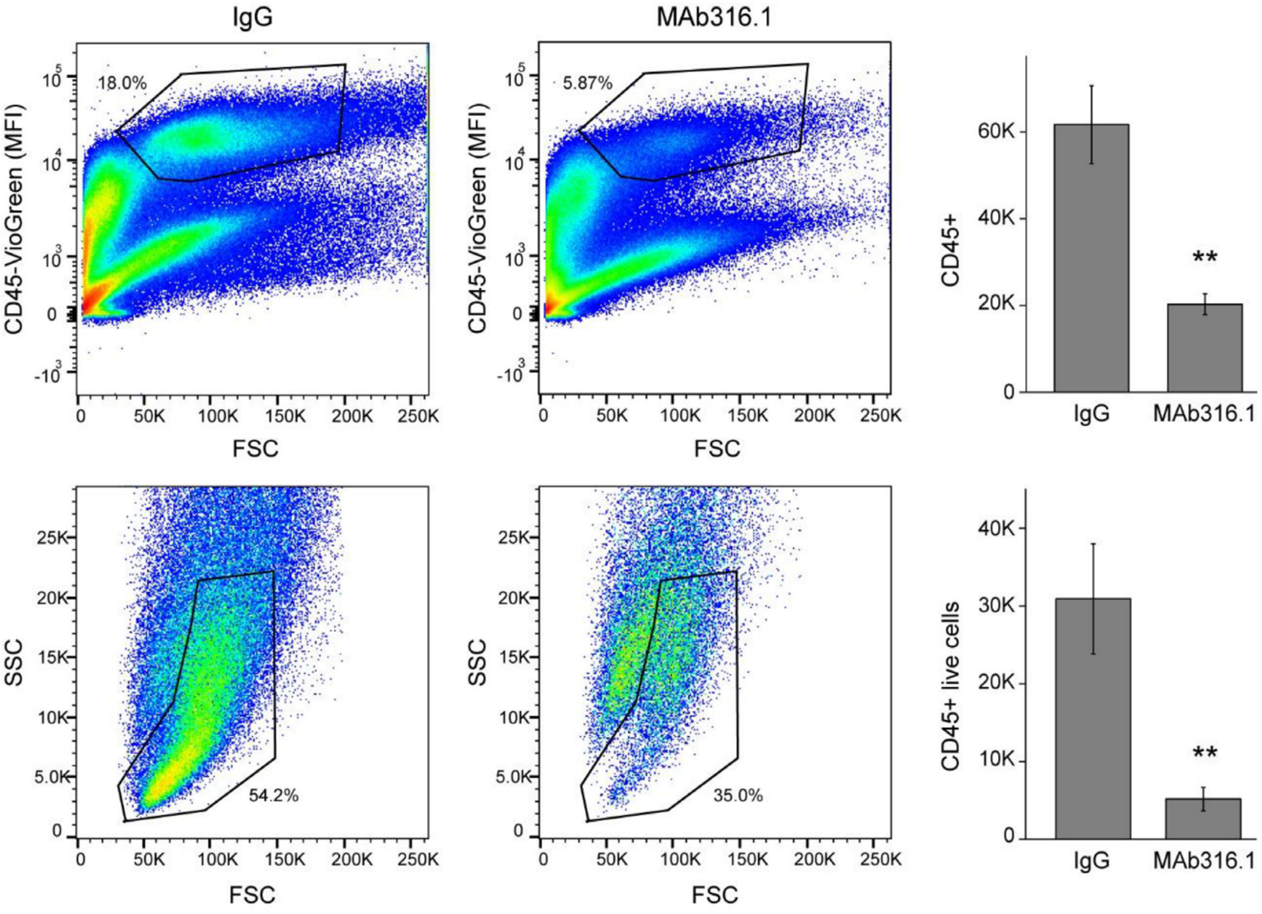

Supplementary Figure 4: FACS analysis of infiltrating immune cells. Upper FACS plots present CD45 labeled cells from representative tumors treated with control IgG or MAb316.1. The positive population is gated in the black polygon. MFI is mean fluorescence intensity. The bar graph on the upper right shows the average numbers of CD45+ recordings for all tumors in each treatment group. CD45+ cells were further analyzed by forward and side scatter as shown in the lower FACS plots. Live cells are gated in the black polygon. The bar graph on the lower right shows the average numbers of live cells for all tumors in each treatment group. The MAb316.1-treated sample had fewer live cells and more aggregates and cell debris. Error bars represent standard error. Asterisks indicate *p*-value compared to IgG control ($^{**} < 0.01$).
